# Experience of Preimplantation Genetic Diagnosis with HLA Matching at the University Hospital Virgen del Rocío in Spain: Technical and Clinical Overview

**DOI:** 10.1155/2014/560160

**Published:** 2014-04-24

**Authors:** Raquel María Fernández, Ana Peciña, Maria Dolores Lozano-Arana, Beatriz Sánchez, Jordi Guardiola, Juan Carlos García-Lozano, Salud Borrego, Guillermo Antiñolo

**Affiliations:** ^1^Department of Genetics, Reproduction and Fetal Medicine, Institute of Biomedicine of Seville (IBIS), University Hospital Virgen del Rocío/CSIC/University of Seville, Avenida Manuel Siurot s/n, 41013 Seville, Spain; ^2^Centre for Biomedical Network Research on Rare Diseases (CIBERER), Seville, Avenida Manuel Siurot s/n, 41013 Seville, Spain

## Abstract

Preimplantation genetic diagnosis (PGD) of genetic diseases, combined with HLA matching (PGD-HLA), is an option for couples at risk of transmitting a genetic disease to select unaffected embryos of an HLA tissue type compatible with that of an existing affected child. Here we present the results of our PGD-HLA program at the Department of Genetics, Reproduction and Fetal Medicine of the University Hospital Virgen del Rocío in Seville. Seven couples have participated in our program because of different indications. Overall, 26 cycles were performed, providing a total of 202 embryos. A conclusive molecular diagnosis and HLA-typing could be assured in 96% of the embryos. The percentage of transfers per cycle was 26.9% and the birth rate per cycle was 7.7% per transfer. Our PGD-HLA program resulted in the birth of 2 healthy babies, HLA-identical to their affected siblings, with successful subsequent haematopoietic stem cell (HSC) transplantations. Both HSC-transplanted children are currently doing well 48 and 21 months following transplantation, respectively. All the procedures, including HSCs umbilical cord transplantation, were performed in our hospital.

## 1. Introduction

The human leukocyte antigen (HLA) system is the name of the major histocompatibility complex (MHC) in humans. The superlocus resides on chromosome 6 and contains a large number of genes that encode cell-surface antigen-presenting proteins that, among several functions, play a major role in the immune system function in humans. Diversity of HLAs in the human population is one aspect of disease defense, and, as a result, the chance of two unrelated individuals with identical HLA molecules on all loci is very low. HLA genes have historically been identified as a result of the ability to successfully transplant organs between HLA-similar individuals. In other words, HLA complex is responsible for rejection following organ/tissue transplantation. Haematopoietic stem cell transplantation (HSCT) from an HLA-identical donor is the best therapeutic option for genetic diseases affecting the haematopoietic and/or immune system in children (e.g., *β*-thalassemia, Fanconi anaemia, etc.) and may be a therapeutic option for acquired diseases (e.g., leukaemia, acquired medullary aplasia, etc.) as well [1, 2]. The frequent unavailability of HLA-identical donors for affected children within the corresponding families or in HSC banks has made the combination of in vitro fertilization (IVF) with HLA-typing for the selection of HLA-identical embryos, a therapeutic approach for these affected children. Moreover, in case of the genetic cause of the disease affecting the children, preimplantation genetic diagnosis (PGD) combined with HLA-typing has emerged as a tool for couples to select unaffected embryos of an HLA tissue type identical to that of an existing affected child [3]. At delivery, HSC from the newborn umbilical cord blood can be used to treat the affected sibling. This approach, firstly applied for Fanconi anaemia by Verlinsky et al. in 2001, is valuable for life-threatening disorders that require an HLA-compatible HSC donor, where HLA identity seems to provide the best chance of avoiding graft rejection and other serious complications of bone marrow transplantation. HLA-typing on one cell is complex because the HLA locus is highly polymorphic and large (4 Mb) and recombination within the locus has been observed [4, 5]. Worldwide, current HLA testing on preimplantation embryos is usually performed using STRs, since multiple STRs throughout the HLA region allow 100% accuracy HLA-typing and detect possible recombination events, as well as the copy number of chromosome 6 [6, 7]. The Centre for Medical Genetics UZ Brussel was the first to report a novel approach for HLA-typing using four evenly distributed informative STRs in multiplex PCR on single cells [6]. Since then, several methodological approaches have been reported with the aim of developing flexible and reliable methodologies for PGD-HLA molecular analyses. Nevertheless, to date a limited number of cases with successful pregnancies and births of healthy HLA compatible donors for patients have been reported [8–18]. Here we present the results of our program of preimplantation HLA-typing, alone or in combination with PGD.

## 2. Materials and Methods

### 2.1. Protocol for the Inclusion of Couples in Our PGD-HLA Program and Ethical Approval

Since 2007, a total of 12 couples have been attended in our department whose reason for consultation was the inclusion in our PGD-HLA-typing program. All of these couples had children affected by either a genetic or an acquired disease affecting their haematopoietic and/or immune system. During the first consultation, the couples provide a detailed medical report from the specialist (generally a hematologist) with an evaluation of the clinical status of the disease in their child, indicating if HSCT is either a consolidated or an experimental therapeutic option for such specific case. In addition, the application of the inclusion in our program must be accompanied by the justification of the lack of matched family donors and, if indicated, the unavailability of other nonrelated matched donors in our National Marrow Donor Registry (Registro de Donantes de Médula Ósea, REDMO). Express agreement of a Service specialized in HSCT must also be included in the documentation, warranting the transplantation of cells from the newborn umbilical cord blood to his affected sibling, in case of success of the PGD-HLA procedure. In addition, in case of a genetic disease, a clear and accurate genetic test report of the corresponding disease for the affected child must be also provided. Extensive genetic counselling and information about the PGD procedures, success rate and possibility of misdiagnosis inherent to techniques, are then given by our multidisciplinary team of geneticists, embryologists, and gynaecologists to the couple.

Informed consent concerning PGD and related procedures as well as the fate of the nontransferred embryos must be signed by the couples. Then, a basic test is prescribed to evaluate the reproductive state of the couples, which includes a hormone analysis and transvaginal ultrasound in the female, seminogram in the male, and serology for hepatitis B and C, HIV, and syphilis in both of them. The results of this test, together with all the documentation previously mentioned, is then sent to our Health Authority (Government of Andalucia) with the aim to obtain its authorization to conduct the PGD-HLA. A prior favorable ruling from our National Commission of Human Assisted Reproduction (Comisión Nacional de Reproducción Humana Asistida, CNRHA), which evaluates the social, therapeutic, and clinical characteristics of each case, is a requisite to obtain the final authorization.

### 2.2. Couples Treated for HLA-Typing Alone or in Combination with PGD

To date a total of seven out of the twelve couples have been authorized to be treated in our PGD-HLA program because of different indications (Table 1). One couple was dismissed; two couples are still pending of the final decision of our health authority, and for the other two couples all the documentation required for evaluation is being currently compiled.

Two out of the seven couples required exclusively HLA-typing to select HLA-matched embryos for their children, who were affected by acquired severe bone marrow aplasia and by a* de novo* mutation related to diamond-blackfan anemia (OMIM#105650), respectively. Regarding the remaining five couples, HLA-typing in combination with PGD was required, with *β*-thalassemia (OMIM#613985) as the indication for 4 of them and adenosine deaminase immunodeficiency (ADA, OMIM#102700) in another one.

### 2.3. Selection of Markers for the Genetic Analyses

Informativity testing for segregation analyses is always developed on the DNA samples from the corresponding family members (father, mother, and affected child) using standard PCR protocols, to identify the “disease haplotypes” and the specific HLA combinations carried by the affected children in the context of their corresponding families.

A first selection of up to 10 STRs was initially made according to their localization along the HLA locus. The policy is to select, for the subsequent PGD, the maximum number of informative STR markers evenly spaced throughout the HLA complex to obtain an accurate haplotyping, allowing identification of double recombination events, which if not detected may lead to misdiagnosis in HLA-typing. Using this panel, we achieved the first successful PGD with HLA-typing performed in Spain [16]. Subsequently, and following the ESHRE PGD guidelines [19], the method has been updated with the inclusion of a selection of another 10 markers along the HLA locus [16].

A panel of six polymorphic short tandem repeats (STRs) located in the neighbouring regions to the **β*-globin* gene was selected to test the status for *β*-thalassemia [16]. Regarding the ADA, we selected 2 STRs surrounding the* ADA* gene (*D20S55* and* D20S16*) and other 2 intragenic STRs located in intron 3 and intron 8, respectively. In each case, the selection was based in the heterozygosity values (>30%) detected for each marker in a group of 30 normal controls, and in their specific location with respect to the genes responsible for the disease, warranting the possibility to detect any recombination event.

### 2.4. Assisted Reproductive Techniques and Embryo Biopsy 

Controlled ovarian stimulation is performed through a long protocol as previously described [20]. Oocytes are carefully denuded from cumulus cells and intracytoplasmic sperm injection (ICSI) is used to prevent contamination with residual sperm adhered to the zona pellucida [19, 21]. Blastomere biopsy is performed on the morning of day three after fertilization. Laser technology (Octax Laser) is used to create an opening in the zona pellucida and one blastomere is gently aspirated for each embryo. Cells are transferred into thin-walled 0.2 mL PCR tubes containing 2.5 *μ*L of lysis buffer and frozen at −80°C before cell lysis.

### 2.5. Multiple Displacement Amplification (MDA) on Single Cells

We adapted the protocol described by Kumar et al. in 2008 [22] to obtain whole genome amplification (WGA) of the blastomeres biopsied from the embryos resulting from the PGD-HLA cycles of the couple with the child affected by ADA. Optimal cell lysis protocol and MDA conditions were set up on single cells biopsied from supernumerary IVF embryos not suitable for transfer or cryopreservation. Efficiency of the MDA protocol on single cells was tested by measurement of absorbance, at *λ* = 260 nm, and the performance of different multiplex PCR protocols on the MDA products.

After blastomeres biopsy, cells are transferred into thin-walled 0.2 mL PCR tubes containing 2.5 *μ*L of lysis buffer [600 mM NaOH, 10 mM EDTA, and 100 mM dithiothreitol (DTT)] and frozen during at least 30 minutes at −80°C before cell lysis. Cell lysis is carried out for 10 min at 65°C, followed by the addition of 1.5 *μ*L neutralizing buffer (Tricine 200 mM, pH = 4.93). In addition, 4 *μ*L sample buffer, 9 *μ*L reaction buffer, and 1 *μ*L enzyme mixture supplied with the Illustra GenomiPhi V2 DNA Amplification kit (GE Healthcare Life Sciences) are added to complete the reaction. The amplification is then carried out at 30°C for 4 h followed by heat inactivation at 65°C for 10 min. Subsequently absorbance of the MDA products at *λ* = 260 nm is measured, and proper dilutions are prepared to obtain aliquots at a final DNA concentration of 25 ng/*μ*L.

### 2.6. Multiplex PCR Protocol on Either Single Cells Or MDA Products

A one-step multiplex single-cell fluorescent PCR is used for the simultaneous amplification of several combinations of markers at the HLA locus alone or in combination with the *β*-globin locus, using the QIAGEN Multiplex PCR kit (QIAGEN, GmbH; Hilden, Germany) and a protocol previously described [16, 23]. Primer sequences and PCR conditions for HLA-typing in combination with PGD for *β*-thalassemia have been previously described [16].

In the case of ADA, a multiplex fluorescent PCR is used for the amplification of a combination of markers linked to the* ADA* locus, in a separate reaction of that used for the HLA typing, using 25 ng of the MDA product in each case. Primers for specific amplification of the ADA-linked markers (available on request) were designed to have a melting temperature of around 55°C, so that the corresponding fragments could be successfully amplified with the same PCR program that the one used for HLA-typing [16].

The different multiplex PCR products are analyzed on an ABI3730 automated sequencer (Applied Biosystems, Foster City, CA).

Prior to the analyses it was established that embryos showing monosomy, trisomy, or uniparental disomy of the chromosomes analyzed would be considered to be abnormal. The embryos with a recombination pattern at the HLA locus are considered to be HLA-nonidentical and therefore not suitable for transfer.

## 3. Results

### 3.1. Diagnosis and HLA-Typing of the Embryos

After analysis of the STR markers for *β*-globin/ADA and/or HLA haplotypes in the context of each family, a specific panel of markers was selected to be further used in the diagnosis/HLA-typing of the embryos resulting from the cycles. The selection of such STRs was made according to their amplification efficiency at the single-cell level, the informativity in the family, and their localization along the tested loci.

At the start of the PGD-HLA program, two cells were taken from each embryo in order to verify the results, but once we experienced that a conclusive and reliable diagnosis for the embryos could be obtained on the basis of one cell, we limited to one cell per embryo.

The percentage of cells with no amplification was 6.0%, which leads to a 3.9% of embryos being undiagnosed, based on the result of at least one cell with PCR amplification. Allele drop-out occurs when a sample is typed and one or more alleles (but not all) are not present, in contrast with what one could expect in the case of monosomy. By the previous segregation analysis in the context of each family, we know the specific combinations of markers associated or not to the disease, as well as the specific HLA-identical haplotypes. Moreover, we specifically select the informative markers to have the warranty to appropriately select embryos. In our experience reported here, we have not detected any ADO for the markers selected in each case. Contaminations were not detected either. Abnormal embryos with monosomies, trisomies, or uniparental disomy comprised 4.6% of cases. Taking into account exclusively the embryos with a conclusive diagnosis for HLA, the global percentage of HLA-identical embryos was 8.8%, and the percentage of HLA-identical unaffected embryos in case of PGD was 8.4% (Tables 1 and 2, and see supplementary table in Supplementary Material available online at http://dx.doi.org/10.1155/2014/560160). A total of 6 embryos showed recombination within the HLA locus (3.0%).

The remaining unaffected embryos resulting from all the cycles that did not achieve enough quality to be cryopreserved (46 embryos), as well as the 50 affected embryos, were retested for the corresponding markers in each case, and the initial results were confirmed in all of them. A total of 42 unaffected and/or non HLA-identical embryos suitable to be cryopreserved were vitrified using the VitKit Freeze kit (Irvine Scientific) and the protocol provided by manufacturers.

### 3.2. Clinical Results

The results of the clinical HLA or PGD-HLA cycles for the seven couples are shown in Table 2. Comparison with the clinical results of other Centers is shown in Table 3. As indicated, a total of 26 cycles were performed, accounting for 5 cycles of exclusively HLA-typing (19.2%) and 21 cycles of PGD-HLA (80.8%). The percentage of mature oocytes suitable to be submitted to ICSI procedures was 77.2%. The fertilization rate, considering the correctly fertilized oocytes out of the total number of mature injected oocytes, was 59.9%. Finally, the overall number of embryos analysed per cycle (88.2% of the embryos) was very variable ranging from 8 to 30 and generally depending exclusively on the couple treated.

Overall 9 embryos were transferred in 7 out of the 26 cycles, which corresponds to a transfer rate of 26.9% (Table 2 and supplementary table).

Of note, in two of the seven couples, efforts resulted in respective pregnancies, with the birth at term of healthy children whose cord blood hematopoietic stem cells were obtained and frozen for a subsequent successful HSCT to their affected siblings. All the procedures, including HSCT, were performed at the University Hospital Virgen del Rocío (HUVR) in Seville. Taking into account these 2 cases, both the clinical pregnancy and live birth rates were 7.7% per cycle and 28.6% per transfer. One of those cases was the first successful case of PGD-HLA in Spain, previously published [16]. The second case corresponds to the couple with a son affected by acquired severe bone marrow aplasia. In this case two HLA-typing cycles were necessary to obtain a successful pregnancy, with the birth at term of a healthy girl. Cord blood HSC was obtained and frozen for later use. The stem cells number in the cord blood was high and HSCT was performed 3 months later. The child is currently doing well and off all treatments 21 months following transplantation.

In summary, 7 couples were treated in 26 cycles and 2 healthy HLA-matched babies were born, leading to a live birth rate of 28.6% per transfer and of 7.7% per initiated cycle.

## 4. Discussion

Verlinsky and collaborators described the first case of PGD-HLA-typing in 2001 [3]. A PGD for Fanconi anaemia in combination with HLA testing was performed to give birth to an unaffected HLA matching sibling. The successful haematopoietic reconstitution in the affected child by HSCT from the HLA-matched offspring was described later [24]. After that, several successful HSC transplantations for genetic and acquired diseases have been reported [8–12, 16–18], representing one of the most relevant achieved challenges in reproductive medicine.

HLA-typing in combination with PGD is a practice allowed only in a few European countries and since 2006 also in Spain. The first Spanish law regulating assisted reproduction in Spain dates back to 1988 (Law 35/1988, of November 22nd). Given the experimental stage of PGD in such date, the regulation of the technique was left to future legislative interventions, but the absence of a specific regulation characterized the Spanish IVF regime until the 2006 Assisted Reproduction Act (Law 14/2006, of May 26th). This law did not establish a closed list of genetic conditions but framed PGD in broader terms in order to introduce a more flexible regulatory regime and to accommodate future technological advances and new genetic conditions without the need to modify the normative framework. The 2006 Act regulated PGD in very permissive terms, supporting the use of this technique not only to avoid the transmission of diseases for which no treatment existed, but also for the selection of embryos for HLA matching. In this specific regard (PGD-HLA matching), the law stipulates a number of conditions, including that cases have to be approved by the National Committee for Assisted Human Reproduction on a case-by-case basis after evaluating the clinical and therapeutic characteristics and weighing carefully the potential risks and benefits to all those involved. As a general rule, preimplantation genetic testing techniques are not paid by public healthcare, although IVF techniques are subsidized in the majority of the regional healthcare systems. In 2005, the Andalusia Regional Government authorized PGD to avoid the implantation of an embryo presenting a genetic profile related to a closed list of specific monogenic diseases (156/2005 decree, of June 28th). For these conditions, IVF and PGD became accessible through the public healthcare system through the HUVR in Seville, one of the leading centers for genetic-based research in Spain. Moreover, since 2006, PGD-HLA was also accessible through our hospital, making it the only public healthcare institution in Spain providing this service.

In our study, the percentage of mature oocytes submitted to ICSI was 77.2%, quite similar to those reported by other centers (Table 3). However, the rate of fertilization was some lower than in other institutions, although it was the same that the general fertilization rate obtained in our hospital for ICSI procedures with exclusively reproductive aims.

In general, the success rate in accurate genetic analyses is quite good, since 96% of the embryos got a correct HLA or PGD/HLA diagnosis (96.3% and 95.2% of the analyzed embryos for PGD/HLA and for HLA, resp.). Initially, 2 cells were biopsied from each embryo to perform the analyses (9 cycles accounting for a total of 88 embryos, Table 1). However, 2 important technical innovations allowed us to reduce the number of biopsied cells to 1 per embryo. The first one was the optimization of a one-step multiplex PCR-based method for HLA-typing and preimplantational genetic diagnosis of *β*-thalassemia. The advantage of such method is that it involves only a round of single PCR for multiple markers amplification (up to 10 markers within the HLA and 6 markers at the *β*-globin loci), leading to a current genotyping success rate of 100% [16]. The second one was the use of MDA as a tool for WGA of the cell, which let us obtain enough DNA quantity to perform a wide spectrum of independent genetic analyses and achieve an accurate molecular diagnosis and HLA-typing, without ADO events among other advantages. Comparative studies suggest that MDA-based WGA procedures produce amplified DNA which is more suitable for a wide range of genetic analysis than DNA from PCR-based WGA methods [22]. This is due to the relatively unbiased amplification by *φ*29 DNA polymerase and the high molecular weight of the amplified DNA compared with PCR-based methods. In fact, MDA had been previously applied to either PGD or PGD-HLA-typing [25] giving satisfactory results, as in our case.

Also worth of note is that the percentage of transfers in our institution (26.9%) is lower than in other centers [7, 9, 12]. This rate is mainly due to the low number of HLA identical embryos obtained per cycle. As shown in Table 1, only 4 out of the 42 embryos for exclusively HLA-typing (9.5%) and 13 out of the 160 embryos for PGD/HLA (8.1%) were HLA-identical, in contrast with the theoretically expected rate of 25%. Moreover, 3 of the 7 couples (2 for PGD/HLA and 1 for HLA only) have not had any transfer yet, although a detailed inspection shows that in those particular cases the response to ovarian stimulation was not good, leading to low figures of oocytes retrieved, mature oocytes injected, fertilized oocytes, and analyzed embryos. This fact obviously affects the percentage of clinical pregnancies per initiated cycle, also relatively lower than in other institutions [7, 9, 12], although the clinical pregnancies per transfer is similar to those previously reported elsewhere.

Finally, although the live birth rate per cycle is slightly lower than other previously reported (7.7%), it is important to note that 2 out of the 7 couples (28.6%) resulted with successful pregnancies and deliveries of HLA-matched embryos. Moreover, HSCT, also performed in our hospital, was successful in both cases (100%).

## 5. Conclusions

The balance of our PGD/HLA program during this period is therefore quite satisfactory, and our results have constituted a relevant advance in the Spanish Public Health system, converting our institution into a referral centre for this therapeutic intervention in our country.

## Supplementary Material

Here we present the figures and/or percentages of our results of genotyping for all the cycles performed for the 7 couples included in our PGD-HLA Program.

## Figures and Tables

**Table 1:** Results of the HLA or PGD/HLA cycles performed in HUVR.

Couple	Mode of transmission	Theoretical probability of suitable embryos	Cycle	Oocytes retrieved	Mature oocytes injected	Fertilized oocytes	Analyzed embryos	Diagnosed embryos	Genetically suitable embryos*	Transferred embryos	Pregnancy/Clinical pregnancy
1 (*β*-THALASSEMIA)	Autosomal Recessive	3/16 (18.75%)	*1 *	20	17	14	11	11	1	1	No
			*2 *	25	22	18	17	17	2	1	Yes/Yes
			Overall	**45**	**39 (86.6%)**	**32 (82%)**	**28 (87.5%)**	**28 (100%)**	**3 (10.7%)**	**2**	

2 (*β*-THALASSEMIA)	Autosomal Recessive	3/16 (18.75%)	1	9	5	5	0	0	0	0	—
			2	9	2	2	2	2	0	0	—
			3	10	6	5	5	5	0	0	—
			Overall	**28**	**13 (46.4%)**	**12 (92.3%)**	**7 (58.33%)**	**7 (100%)**	**0 (0%)**	**0**	**—**

3 (ADA)	Autosomal Recessive	3/16 (18.75%)	*1 *	29	23	12	9	9	0	0	—
			*2 *	19	17	12	12	11	0	0	—
			*3 *	23	20	9	9	9	0	0	—
			4	18	18	12	8	8	2	0	—
			5	17	14	11	10	9	0	0	—
			Overall	**106**	**92 (86.8%)**	**56 (60.87%)**	**48 (85.7%)**	**46 (95.83%)**	**2 (4.4%)**	**0**	

4 (*β*-THALASSEMIA)	Autosomal Recessive	3/16 (18.75%)	*1 *	11	11	4	4	4	0	0	—
			*2 *	19	13	7	7	7	0	0	—
			*3 *	20	16	8	8	5	0	0	—
			*4 *	29	22	12	11	11	1	1	No
			5	18	16	9	8	8	0	0	—
			6	14	11	7	7	6	1	1	Yes/No
			7	18	14	9	7	7	1	0	—
			8	29	15	7	6	6	0	0	—
			9	30	27	10	10	10	4	2	No
			Overall	**188**	**143 (76.0%)**	**73 (51.1%)**	**68 (93.2%)**	**64 (94.1%)**	**7 (10.9%)**	**4**	

5 (*β*-THALASSEMIA)	Autosomal Recessive	3/16 (18.75%)	1	8	4	3	3	3	0	0	—
			2	16	14	6	6	6	0	0	—
			Overall	**24**	**18 (75%)**	**9 (50%)**	**9 (100%)**	**9 (100%)**	**0 (0%)**	**0**	**—**

6 (BONE MARROW APLASIA)	Acquired	1/4 (25%)	1	29	28	14	14	13	3	2	No
			2	34	27	19	19	18	1	1	Yes/Yes
			Overall	**63**	**45 (71.4%)**	**33 (73.3%)**	**33 (100%)**	**31 (93.9%)**	**4 (12.9%)**	**3**	

7 (DIAMOND-BLACKFAN ANEMIA)	De novo	1/4 (25%)	1	12	9	4	4	4	0	0	—
			2	14	11	6	3	3	0	0	—
			3	15	12	4	2	2	0	0	—
			Overall	**41**	**32 (78.0%)**	**14 (43.8%)**	**9 (62.3%)**	**9 (100%)**	**0 (0%)**	**0**	

Cycles in which molecular diagnosis and HLA-typing were performed on 2 cells biopsied from each embryo, are in italic. For the remaining cycles, just 1 blastomere was biopsied per embryo.

*“Genetically suitable embryos” were those non-affected embryos that were HLA-identical to the affected patient within the context of each family.

**Table 2:** Clinical data for preimplantational HLA typing at HUVR.

	HLA + PGD	HLA-only	Total
No of couples treated	5	2	7
Maternal age	31.0 ± 2.4	28.0 ± 2.8	30.1 ± 2.7
No of cycles performed	21	5	26
No of cycles performed per couple	4.2 ± 2.9	2.5 ± 0.7	3.7 ± 2.6
No of oocytes retrieved	391	104	495
No of oocytes retrieved per cycle	18.6 ± 7.0	16.3 ± 9.0	17.9 ± 7.6
No of mature oocytes submitted to ICSI	305	77	382
% of oocytes injected	78.0%	74.0%	77.2%
No of mature oocytes submitted to ICSI per cycle	14.6 ± 6.5	13.1 ± 9.3	14.2 ± 7.3
No of oocytes fertilized	182	47	229
% of oocytes fertilized	59.7%	61.0%	59.9%
No of oocytes fertilized per cycle	8.7 ± 3.9	7.3 ± 5.5	8.3 ± 4.4
No of embryos analyzed	160	42	202
% of embryos analyzed	87.9%	89.4%	88.2%
No of embryos analyzed per cycle	7.6 ± 3.8	6.2 ± 6.4	7.2 ± 4.6
No of transfers	5	2	7
% of transfers	23.8%	40%	26.9%
No of embryos transferred	6	3	9
No of pregnancies	2	1	3
No of clinical pregnancies	1	1	2
No of clinical pregnancies per cycle	4.8%	20%	7.7%
No of clinical pregnancies per transfer	20%	50%	28.6%
No of embryos implanted	2	1	3
Implantation rate	33.3%	33.3%	33.3%
No of pregnancies went to term	1	1	2
No of babies born	1	1	2
Live birth rate per cycle	4.8%	20%	7.7%

**Table 3:** Comparison of the clinical data for preimplantational HLA typing at different Centres.

	Reproductive Genetics Institute Chicago (Rechitsky et al., 2004) [7]	UZ Brussel (Van de Velde et al., 2009) [12]	Genoma (Van de Velde et al., 2009) [12]	Istanbul Memorial Hospital's (Kahraman et al., 2011) [9]	HUVR, Spain (this work)
No of couples treated	26	32	107	171	7
No of cycles performed	46	85	199	327	26
% of oocytes injected	NA	82.7%	76.8%	NA	77.2%
% of oocytes fertilized	NA	68.0%	88.5%	NA	59.9%
% of embryos analyzed	NA	40.3%	76.5%	NA	88.2%
% of embryos diagnosed	93.0%	99.1%	94.2%	92.0%	96.0%
No of transfers	33	27	138	NA	7
% of transfers	71.7%	31.8%	69.3%	64.8%	26.9%
No of embryos transferred	50	34	216	NA	9
No of clinical pregnancies	6	9	48	NA	2
% of clinical pregnancies per cycle	13.0%	10.6%	24.1%	NA	7.7%
% of clinical pregnancies per transfer	18.2%	33.3%	34.8%	34.9%	28.6%
Implantation rate	12.0%	32.4%	28.7%	26.3%	33.3%
No of pregnancies went to term	5	8	37	52	2
Live birth rate per cycle	10.9%	9.4%	18.6%	15.9%	7.7%
