# Supplementary material for: Experience of Preimplantation Genetic Diagnosis with HLA Matching at the University Hospital Virgen del Rocío in Spain: Technical and Clinical Overview
Source: Biomed Res Int. 2014 Apr 24;2014:560160. doi: 10.1155/2014/560160 (PMC4017834; doi:10.1155/2014/560160)
Supplement: Supplementary file 1 — Here we present the figures and/or percentages of our results of genotyping for all the cycles performed for the 7 couples included in our PGD-HLA Program. [file 560160.f1.pdf]

Supplementary Table: Results of genotyping at HUVR

	HLA+PGD	HLA-only	Total
No of embryos analyzed	160	42	202
No of embryos diagnosed	154	40	194
% of embryos diagnosed	96.3	95.2	96.0
No of HLA identical embryos	13	4	17
% of HLA identical embryos	8.1	9.5	8.4
No of HLA identical healthy embryos	10	-	10
% of HLA identical healthy embryos	6.3	-	6.3
No of affected embryos	50	-	50
No of non affected embryos	97	-	97
No of abnormal embryos	7	2	9
% of abnormal embryos	4.5	5.0	4.6
No of embryos with monosomy	6	2	8
No of embryos with trisomy	-	-	-
No of embryos with uniparental disomy	1	-	1
